# A Rapid Single-Cell Antimicrobial Susceptibility Testing Workflow for Bloodstream Infections

**DOI:** 10.3390/bios11080288

**Published:** 2021-08-22

**Authors:** Britney Forsyth, Peter Torab, Jyong-Huei Lee, Tyler Malcom, Tza-Huei Wang, Joseph C. Liao, Samuel Yang, Erik Kvam, Chris Puleo, Pak Kin Wong

**Affiliations:** 1 Department of Biomedical Engineering, The Pennsylvania State University, University Park, PA 16802, USA; btf5096@psu.edu (B.F.); jkl6028@psu.edu (J.-H.L.); tnm5111@psu.edu (T.M.); 2 Department of Mechanical Engineering, The Pennsylvania State University, University Park, PA 16802, USA; pot5076@psu.edu; 3 Departments of Mechanical Engineering and Biomedical Engineering, Johns Hopkins University, Baltimore, MD 21218, USA; thwang@jhu.edu; 4 Department of Urology, Stanford University School of Medicine, Stanford, CA 94305, USA; jliao@stanford.edu; 5 Department of Emergency Medicine, Stanford University School of Medicine, Stanford, CA 94305, USA; syang5@stanford.edu; 6 GE Global Research, Niskayuna, NY 12309, USA; kvame@ge.com (E.K.); puleo@ge.com (C.P.); 7 Department of Surgery, College of Medicine, The Pennsylvania State University, Hershey, PA 17033, USA

**Keywords:** sepsis, diagnostics, multidrug-resistant bacteria, single-cell analysis, microfluidics

## Abstract

Bloodstream infections are a significant cause of morbidity and mortality worldwide. The rapid initiation of effective antibiotic treatment is critical for patients with bloodstream infections. However, the diagnosis of bloodborne pathogens is largely complicated by the matrix effect of blood and the lengthy blood tube culture procedure. Here we report a culture-free workflow for the rapid isolation and enrichment of bacterial pathogens from whole blood for single-cell antimicrobial susceptibility testing (AST). A dextran sedimentation step reduces the concentration of blood cells by 4 orders of magnitude in 20–30 min while maintaining the effective concentration of bacteria in the sample. Red blood cell depletion facilitates the downstream centrifugation-based enrichment step at a sepsis-relevant bacteria concentration. The workflow is compatible with common antibiotic-resistant bacteria and does not influence the minimum inhibitory concentrations. By applying a microfluidic single-cell trapping device, we demonstrate the workflow for the rapid determination of bacterial infection and antimicrobial susceptibility testing at the single-cell level. The entire workflow from blood to categorical AST result can be completed in less than two hours.

## 1. Introduction

Bloodstream infections (BSI), which cause sepsis, shock, and other life-threatening complications, are major global healthcare challenges [1]. The timely identification of bloodborne pathogens is a recognized clinical bottleneck in the management of BSI [2]. In the current clinical microbiological analysis workflow, vials of blood are drawn from patients (8–10 mL for adult patients). The blood vials are cultured in aerobic and anaerobic conditions for up to five days to detect the presence of pathogens. If the culture is positive, samples from the culture bottles are used for Gram staining and molecular analysis (e.g., polymerase chain reaction) of pathogens to identify the species. The confirmation of a bacterial infection and identification of the bacterial species can facilitate the selection of proper treatment. Moreover, a pathogen-specific treatment can be administrated if the AST result is available. To determine the susceptibility of a bacterium to an antibiotic, a few drops of the blood culture sample are placed in agar media plates, and colonies are grown in the presence or absence of drugs to detect colony growth. An automated machine, such as the Vitek system, is often used for this procedure, which can take an additional 5–10 h. Due to these time-consuming processes to obtain both microbial identification and AST from blood, patients are often prescribed broad-spectrum antibiotics prior to obtaining a precise diagnosis. However, precise antibiotic treatments, in contrast to broad-spectrum antibiotics, are more effective and can minimize the disruption of the commensal microbiota, which improves the clinical outcome [3]. Unfortunately, the prolonged delay in microbiological diagnosis promotes the improper usage of antibiotics, which increases patient mortality and the emergence of antibiotic-resistant pathogens.

Extensive efforts have been devoted to address the need for rapid microbiological analysis [4,5]. In particular, single-cell analysis platforms are highly promising for providing high resolution diagnosis with a quick turnaround time. For example, automated single-cell morphological analysis platforms with machine learning algorithms provide cost-effective and accurate antimicrobial susceptibility data in non-traditional healthcare settings [6,7,8,9]. A nanoarray digital polymerase chain reaction with high resolution melt curve analysis enables rapid broad bacteria identification and phenotypic AST [10,11]. Furthermore, single-cell microfluidic devices, along with molecular biosensors, allow the rapid classification of the pathogen, the detection of polymicrobial samples, the identification of bacterial species, and single-cell AST [12,13,14,15,16]. These platforms have been demonstrated for the rapid diagnosis of various common infections, such as urinary tract infections and wound infections. Due to the low bacteria load, single-cell analysis is particularly attractive for the diagnosis of BSI without the blood tube culture step. Nevertheless, BSI diagnosis remains challenging due to the low bacteria concentration (10^0^–10^1^ cfu/mL) and the complex matrix effect of blood [17]. Sample preparation procedures based on centrifugation and filtering have been developed to isolate bacteria from whole blood [18]. However, the difficult manual steps associated with these techniques and pathogen-species-specific challenges (such as filter interactions or pathogen–host cell interactions) often make the clinical translation of these techniques impractical. Effective sample preparation procedures that bypass the lengthy blood culture step are, therefore, highly sought-after for the single-cell microbiological analysis of BSI.

**Figure 1:**
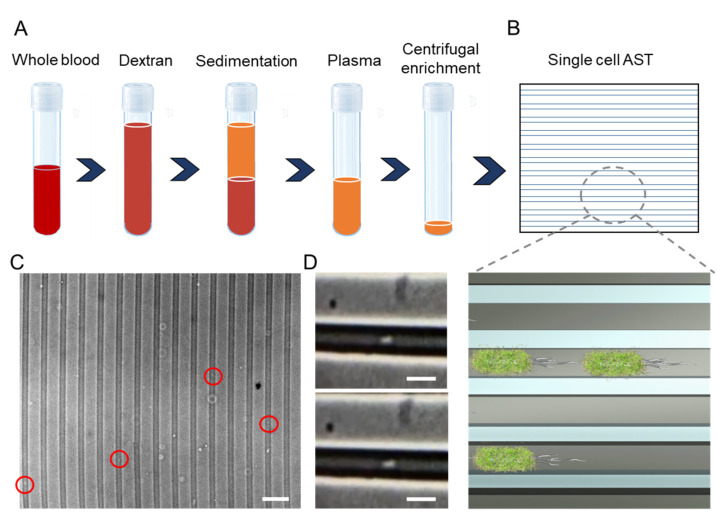
A workflow for rapid bacteria isolation from whole blood for single-cell AST. (**A**) Schematic of the sample preparation workflow with dextran sedimentation and centrifugation for single-cell AST of bloodstream infection. (**B**) Schematic of the microfluidic device for single-cell AST. (Bottom) Zoom-in view of bacteria trapped in the microchannel. (**C**) In the single-cell AST device, bacteria are trapped in the microscale channel for visualizing the presence of bacteria and their response to antibiotics. Scale bar, 20 µm. (**D**) Images of individual bacteria trapped in the microfluidic channel. Scale bars, 5 µm.

Here, we report a culture-free sample preparation workflow for rapid microbiological analysis of BSI (Figure 1A,B). The workflow starts with dextran sedimentation for red blood cell (erythrocyte) depletion, which has been applied for immunological analysis, such as neutrophil purification, and other biomedical applications [19,20]. We evaluate the capability of the dextran sedimentation procedure for depleting red blood cells while maintaining the bacteria in the sample. We also test the efficiency of the procedure for common bacterial pathogens, such as *Escherichia coli*, *Klebsiella pneumoniae*, *Enterococcus faecalis*, and *Staphylococcus aureus*, and optimize the procedure to minimize species-specific bacteria-mediated coagulation [21,22]. The dextran-isolated bacteria are then enriched by centrifugation, providing an effective method for red blood cell depletion compared to the complex selective lysis or gradient centrifugation techniques currently employed [11,23]. The enriched sample is loaded into a microfluidic device for determining the presence of bacteria in the sample and phenotypic single-cell AST (Figure 1C,D). We demonstrate the blood-to-AST workflow in less than 2 h compared to 5–7 days using the standard blood tube culture-based techniques in clinical laboratories.

## 2. Materials and Methods

### 2.1. Sample and Reagents

All reagents were obtained from Sigma (St. Louis, MO, USA) unless otherwise noted. Pathogenic bacteria isolates (*Escherichia coli*, *Klebsiella pneumoniae*, *Enterococcus faecalis*, and *Staphylococcus aureus*) were isolated from patient urine samples under an approved protocol from the Stanford University Institutional Review Board. The antimicrobial resistance profiles for pathogenic *E. coli* were previously determined by the clinical microbiology laboratory at the Veterans Affairs Palo Alto Health Care System. *E. faecium* was obtained from ATCC (ATCC 35667). Human blood samples were purchased from BioIVT in Vacutainer blood collection tubes. These tubes were in 10 mL aliquots and were stored at 4 °C before use.

### 2.2. Bacterial Isolation and Enrichment Workflow

To isolate and enrich bacteria from whole blood, the dextran and sodium polyanethole sulfonate (SPS) solutions were first filtered using a polyethersulfone (PES) membrane with 0.2 µm pore size. The bacterial sample was diluted to 2 × 10^5^ cfu/mL, and the appropriate volume was spiked into the blood solution to control the concentration (10–100 cfu/mL). The mixture contains 10 mL of whole blood, 12 mL of 2.25% 500 kDa dextran solution (Spectrum D1004), and 1.98 mL of 1% SPS solution. The mixture was allowed to sediment at room temperature until a clear plasma-like layer (referred to as the plasma layer below) was formed (~15–30 min). This top plasma layer was removed and mixed with a pipette to ensure the equal distribution of bacteria. The plasma layer was separated into 4 tubes, each containing a volume ~1 mL. Each tube was centrifuged at 2000× *g* for 5 min (Denville 260D Brushless Centrifuge). The upper layer was removed, and the pellet containing bacteria and any human cells not removed in the sedimentation step was resuspended in 0.1 mL of Mueller–Hinton (MH) broth. Bacteria counts were determined by plate counting, and recovery rates were estimated by the portion of recovered bacteria relative to the amount of bacteria spiked into the samples.

### 2.3. Device Fabrication

The microfluidic device for single-cell AST was fabricated by soft lithography. The microchannel master mold was fabricated by photolithography patterning and reactive-ion etching of a silicon wafer. Microchannel layers were then fabricated by polydimethylsiloxane (PDMS) molding on the master mold. PDMS pre-polymer and cross-linker were mixed at 10:1 ratio. The mixture was poured on the master mold and incubated for at least 3 h at 65 °C. The single-cell AST device was fabricated by bonding the PDMS layer with a glass slide. Inlet and outlet reservoirs were created by punching the PDMS layer with a biopsy puncher.

### 2.4. Single-Cell AST

To perform the microfluidic single-cell AST experiment, ampicillin was added to the enriched samples with concentrations of 0 µg/mL, 2 µg/mL, 4 µg/mL, and 8 µg/mL. Each respective solution was loaded into a microfluidic device by capillary force. The devices were then mounted onto an epi-fluorescence microscope (Leica DMI 4000B, objective 20× or 40×) with a microscope heating stage. The presence of bacteria was examined, and the bacterial growth was monitored continuously.

### 2.5. Statistical Analysis

Data analyses were performed with Excel. The data were analyzed using one-way analysis of variance and Tukey’s post-hoc test. Data represent the mean ± s.e.m. A two-sided *p*-value of <0.05 was considered statistically significant.

## 3. Results

### 3.1. Workflow for Bloodstream Infection Analysis

To allow the rapid analysis of pathogens in BSI, we developed a workflow for directly isolating bacterial pathogens in whole blood without the blood culture step (Figure 1A,B). The workflow includes three major steps. First, a dextran solution and SPS are mixed with whole blood. The mixture is then allowed to settle and sediment for <30 min to deplete the red blood cells. The plasma (the upper half or clear portion of the solution) is then pipetted out carefully. Second, the plasma is further enriched by centrifugation. The enrichment step (5 min) is required to reduce the sample volume and increase the concentration of bacteria for microfluidic single-cell analysis. But, with a majority of red blood cells removed by the simple sedimentation step, centrifugation becomes a one-step process to achieve volume reduction, instead of the multi-step process required if using common selective lysis or gradient centrifugation methods for bacteria selection. After the removal of the supernatant, the pellet was resuspended in 50 microliters of MH broth. Third, the sample was loaded into microchannels with cross-sectional dimension compatible to the characteristic length (e.g., width) of the bacteria (Figure 1B). The channel functioned as a filter to separate the sample matrix (cell debris or other cell components) and enabled the visualization and enumeration of bacteria in the sample (Figure 1C,D). The microfluidic channel also trapped the bacteria to facilitate the monitoring of the bacteria response (~ 1 h) to antibiotics (i.e., phenotypic AST).

### 3.2. Efficiency of Dextran Sedimentation

**Figure 2:**
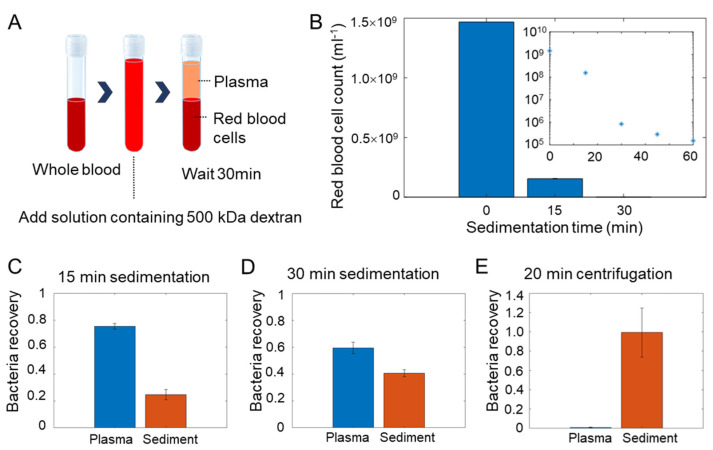
Dextran sedimentation for blood cell removal. (**A**) Schematic of the dextran sedimentation protocol for the removal of blood cells. (**B**) Blood cell removal efficiency. The cell count decreases exponentially with the sedimentation time. Inset, the data are plotted in semi-log scale to illustrate cell removal for several orders of magnitude. (**C**,**D**) Bacteria recovery (portion of total bacteria) with 15 and 30 min of dextran sedimentation. (**E**) Bacteria recovery and sample volume reduction by a soft spin at 200 g for 20 min. Data represent mean ± s.e.m. (*n* = 3).

We first evaluated the red blood cell depletion efficiency of the dextran sedimentation step using *E. coli* (Figure 2A). The concentration of red blood cells was measured as a function of the sedimentation time by cell counting with a hemacytometer (Figure 2B). The initial concentration of red blood cells was on the order of 10^9^ cells per ml. The red cell counts dropped to 10^5^–10^6^ cells per ml in the first 30 min. After that, the blood cell count further reduced at a slower rate (Figure 2B inset). In contrast, the clear portions of the solution after 15 to 30 min of sedimentation retained the majority (>50%) of the amount of the spiked bacteria (Figure 2C,D). We therefore chose to sediment for <30 min in our protocol. Since we recovered ~50% of plasma by volume, the effective concentration of the bacteria in blood (per ml) was similar to the initial concentration, while the majority of red blood cells was removed. This is a substantial enhancement compared to the direct centrifugation of the bacteria. The specific gravities of human red blood cells and *E. coli* are both around 1.08–1.1 g/mL [24,25]. Due to their similarity in specific gravity, direct centrifugation resulted in a considerable loss of bacteria into the sediment portion (Figure 2E). In contrast, the dextran sedimentation step allowed the depletion of red blood cells while maintaining the effective concentration of bacteria in the sample.

### 3.3. Isolation Efficiency for Common Pathogens

**Figure 3:**
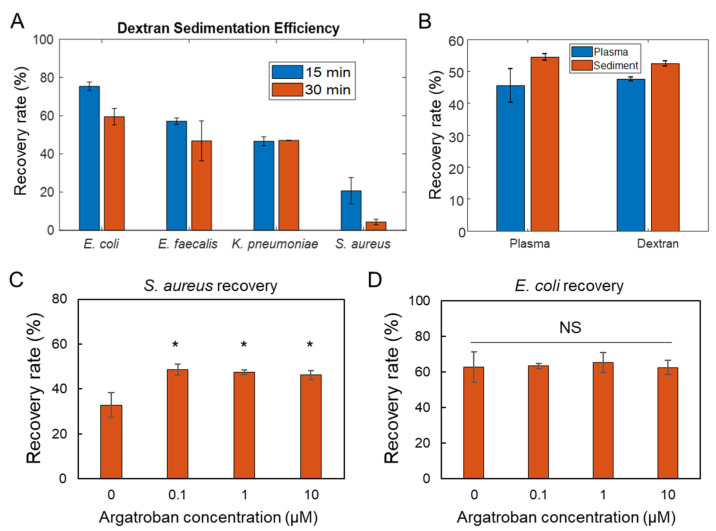
Dextran sedimentation efficiency for common pathogens. (**A**) Recovery rates of *E. coli*, *E. faecalis*, *K. pneumoniae*, and *S. aureus* after dextran sedimentation. (**B**) Control experiments with plasma and dextran solution for characterizing the recovery rate of *S. aureus*. (**C**,**D**) Recovery rates of *S. aureus* and *E. coli* with a thrombin inhibitor, argatroban. The results were obtained after 30 min dextran incubation. Data represent mean ± s.e.m. (*n* = 3). One-Way ANOVA with Tukey’s HSD test. * *p* < 0.05, NS = not significant.

To evaluate the applicability of the dextran sedimentation step for BSI diagnostics, the procedure was performed in human whole blood samples spiked with several clinical bacterial isolates. In particular, the procedure was tested with *E. coli*, *K. pneumoniae*, *E. faecalis*, and *S. aureus* (Figure 3A). These bacteria cover both Gram-negative and Gram-positive species and represent clinically important multidrug-resistant pathogens that cause BSI and other bacterial infections. In the experiment, *E. coli*, *E. faecalis*, and *K. pneumoniae* were recovered with 50–60% efficiency as expected. However, the recovery of *S. aureus* resulted in a lower recovery efficacy and high batch-to-batch variation. The recovery rate was between 10% and 30%, compared to over 50% in other bacteria.

To explore the mechanism responsible for the lower recovery rate of *S. aureus*, the sedimentation step was repeated in isolated plasma (i.e., the majority of blood cells removed) and in buffer (i.e., no blood cells and blood proteins). In both conditions, the plasma and sediment portion had an approximately equal concentration of bacteria (Figure 3B). Therefore, the reduction in recovery rate likely involved both blood cells and plasma proteins (e.g., clotting factors). *S. aureus* is uniquely known to agglutinate in blood and plasma through the action of bacterial clumping factors that interact with host proteins in blood [21,22]. We therefore hypothesized that coagulated *S. aureus* might become passively entangled with red blood cell rouleaux in dextran [26], whereas this would not be an issue in RBC-depleted plasma (as tested in Figure 3B).

To test the hypothesis that the reduced isolation efficiency is a result of *S. aureus*-mediated coagulation, we applied an anticoagulant, argatroban, into the mixture during the dextran sedimentation procedure. The results revealed that the recovery rate was restored to over 50% with 0.1 µM of argatroban (Figure 3C). A higher argatroban concentration did not further improve the recovery, suggesting that a small amount of anticoagulant is sufficient to eliminate the effect of the *S. aureus*-mediated coagulation. The experiment was also performed in *E. coli* to verify that anticoagulant treatment did not influence the dextran sedimentation efficiency in other bacteria. These results suggest the dextran sedimentation step is suitable for isolating common bacterial pathogens, and the addition of argatroban into the sedimentation tube enables the processing of pathogens known to interact with blood cells and the coagulation cascade.

### 3.4. Minimum Inhibitory Concentration (MIC) of Bacteria Isolated from Blood

**Figure 4:**
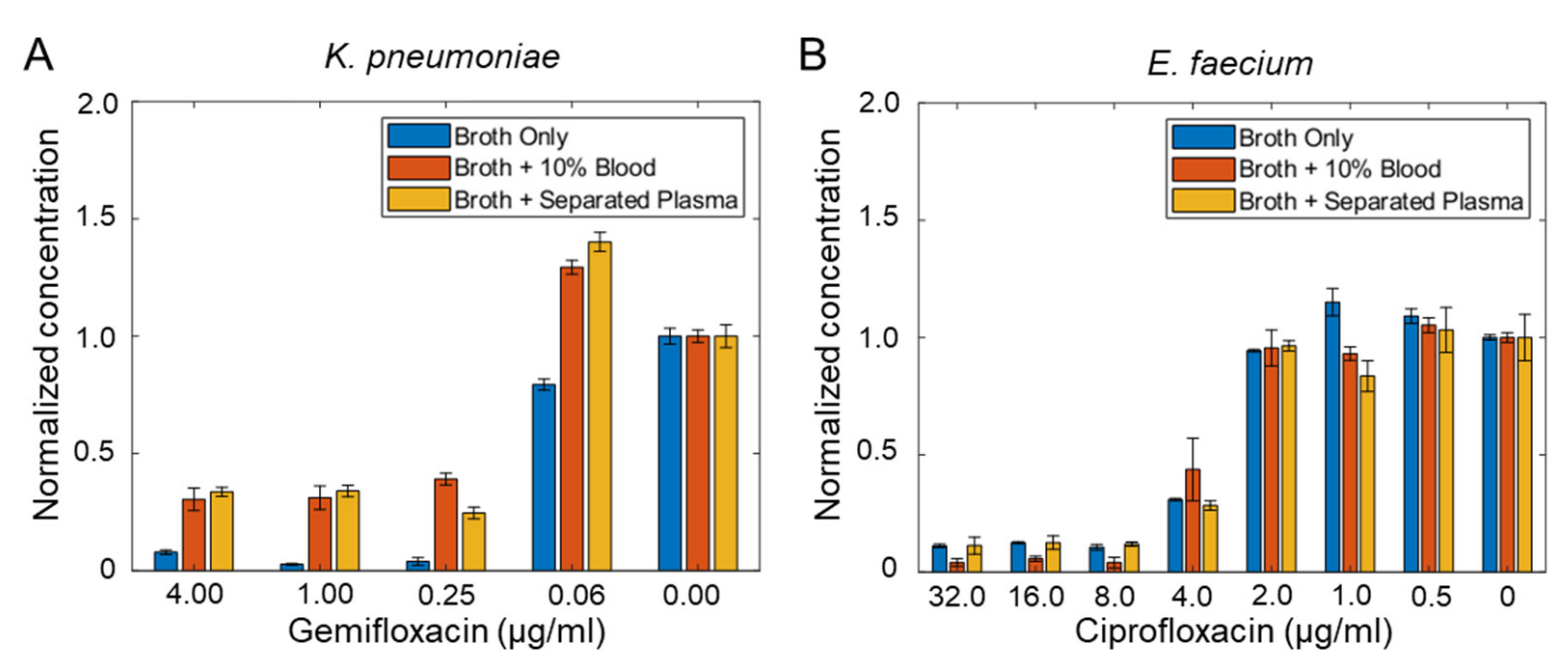
Effect of dextran on minimum inhibitory concentration. (**A**,**B**) Phenotypic growth of (**A**) *K. pneumoniae* and (**B**) *E. faecium* in buffer, 10% blood, and dextran-separated plasma. The experiment was performed using 5 mL blood tubes. The samples were plated on agar plates for approximately 24 h for colony counting. The minimum inhibitory concentration values were not affected by the dextran sedimentation protocol. Data represent mean ± s.e.m. (*n* = 3).

One of the goals of our study is to perform direct AST without the time-limiting blood culture step. To evaluate if dextran and the remaining blood component influence the AST result, we performed AST experiments with broth only, broth with 10% blood, and dextran-isolated plasma with MH broth at 1:1 ratio. The broth-only case represented a standard AST condition. The broth with 10% blood was included to evaluate the influence of blood components (cells and proteins) on the MIC. The separated plasma mixed with MH broth at 1:1 ratio tested the effect of dextran and represented the AST condition in the proposed workflow. The experiment was performed in *K. pneumoniae* and *E. faecium* (Figure 4). Our results suggested the MIC was not affected by the inclusion of 10% blood or dextran separation protocol. For example, the *E. faecium* has a MIC between 4 and 8 μg/mL in all three conditions. These results further support our workflow for direct AST from whole blood samples.

### 3.5. Microfluidic Single-Cell Analysis

A microfluidic device was incorporated for analyzing bacteria in the separated plasma [12,14]. The microchannel assists the visualization of individual bacteria, determines the presence of bacteria, and performs AST phenotypically. However, a challenge of direct blood analysis is the low bacteria concentration (10^0^–10^1^ cfu/mL). Since the microfluidic AST device handles only 5–50 μL of fluid, the effective bacteria count could be less than 1 cfu. Therefore, a centrifugation step was incorporated to enrich the sample through volume reduction. The recovery rate of the centrifugation step was determined to be over 80% based on the plate count method. Enriched samples were then directly loaded into the inlet of the microfluidic devices for bacterial trapping. Since the microchannel height (1.3 µm) was compatible with the size of a bacterium, bigger objects, e.g., blood cells, were effectively filtered out by the channel. Without the dextran sedimentation step, filtering by the microchannel, however, was not possible due to the clogging of the channel by the blood cells. The presence of viable bacteria in the sample was determined by microscope inspection of the motility and growth of the bacteria. We demonstrate trapping of bacteria in blood samples with as low as 10 cfu/mL (Figure 1C,D). Since blood is generally sterile, the presence of bacteria can provide a direct indication of bacterial infection. In previous reports, we have also demonstrated that the multiple channel heights within the microchannel device can be used for size-based classification of the bacteria [12].

**Figure 5:**
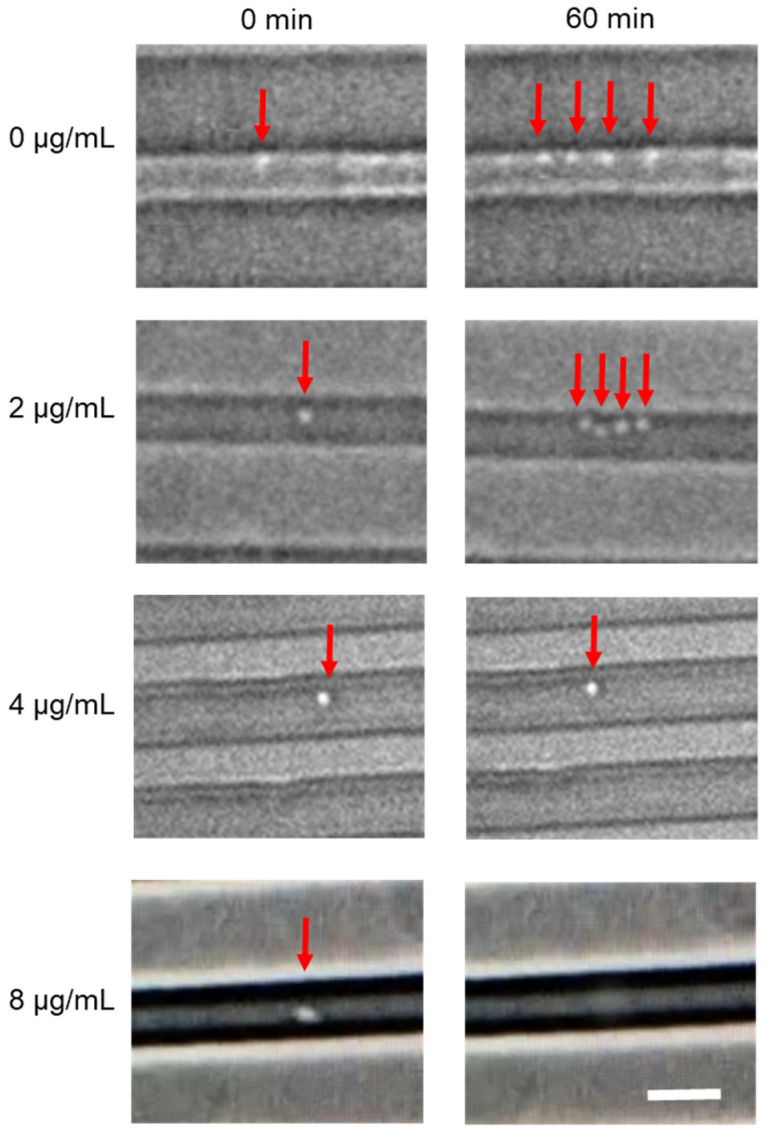
Phenotypic single-cell AST. Growth of isolated *E. coli* in single-cell microchannels with varying concentrations of ampicillin. Red arrows indicate the position of the bacteria. Growth of bacteria was observed at or below 2 µg/mL ampicillin. At 8 µg/mL, the bacterium was lysed. Scale bar, 5 µm.

We further demonstrated the workflow for single–cell AST. The microfluidic device trapped bacteria in one–dimensional channels, and the bacteria were allowed to grow along the channel for phenotypic AST. The antimicrobial susceptibility of an E. coli clinical isolate to ampicillin was tested as a demonstration (Figure 5). The sample was separated into four tubes and mixed with different concentrations of antibiotics. The MIC of the bacteria strain was between 2 and 4 µg/mL. The growth of bacteria was only observed when the concentration of ampicillin was below 2 µg/mL. At a higher concentration (e.g., 8 µg/mL) of ampicillin, which is bacteriolytic, some bacteria were lysed during the duration of the experiment. The MIC value was below the susceptible breakpoint for Enterobacteriaceae according to the Clinical and Laboratory Standards Institute (CLSI) guideline. The result was in categorical agreement with the clinical microbiology laboratory report [14,27]. The data demonstrated the workflow for the rapid diagnostics of BSI from whole blood to AST.

## 4. Discussion and Conclusions

This study reports a culture-free workflow for BSI diagnostics. We demonstrate the workflow for isolating common antibiotic-resistant bacteria from whole blood. This workflow requires relatively simple equipment and procedures, which can be potentially implemented in non-traditional healthcare settings. If the resources (e.g., power) are limited, portable and hand-powered centrifuges can be considered to simplify the system requirement further, as the sedimentation step requires only a single volume-reduction centrifugation step [28,29]. The isolation and enrichment steps could be finished in approximately 30 min, which is similar to or faster than other BSI diagnostic workflow [11,23]. We show single-cell AST using the microfluidic cell trapping device in this study. As shown in our previous study, pathogen classification can be performed in as fast as 5 min by microscopic examination, and AST results can be obtained in a timescale similar to the doubling time of the pathogen [11,23]. The microfluidic device also standardizes the broth volume, which minimizes the influence of the inoculum effect [30], and promotes rapid bacteria growth by facilitating gas exchange [31]. The MIC was also correctly identified using the microfluidic system. Importantly, the workflow maintains the viability of the bacteria and is compatible with other single-cell microbiological analysis platforms, including machine learning-based morphological analyzers and microfluidic molecular assays [7,8,9,10,11,13]. In the future, these techniques can be implemented with the workflow to allow rapid pathogen identification and AST.

In this study, we demonstrate the workflow for single-cell AST at a clinically relevant concentration (10 cfu/mL). Sepsis diagnostics, however, could be as low as 1 cfu/mL. Notably, the isolated sample was separated into multiple tubes for testing various antibiotic conditions. The limit of detection of the workflow can be enhanced by further optimizing the workflow. For instance, the initial blood volume can be enhanced to increase the bacteria count in the sample. If necessary, a short pre-culture step (e.g., 2 h) can be added in the workflow to increase the initial bacteria count. The efficiency of bacteria loading can also be enhanced by incorporating other microfluidic modules (e.g., electrokinetic trapping and enrichment) [14,32]. Future study should also evaluate the workflow using blood samples from patients to evaluate the influence of sepsis-induced effects (e.g., an elevated white blood cell count). Incorporating these changes will enable a new generation of rapid, culture-free BSI diagnostic techniques in the future.

## Data Availability

Not applicable.
